# A Mobile App for the Self-Report of Psychological Well-Being During Pregnancy (BrightSelf): Qualitative Design Study

**DOI:** 10.2196/10007

**Published:** 2018-11-27

**Authors:** Kevin Doherty, Marguerite Barry, José Marcano-Belisario, Bérenger Arnaud, Cecily Morrison, Josip Car, Gavin Doherty

**Affiliations:** 1 School of Computer Science and Statistics Trinity College Dublin Dublin Ireland; 2 School of Information and Communication Studies University College Dublin Dublin Ireland; 3 School of Public Health Imperial College London London United Kingdom; 4 Microsoft Research Cambridge United Kingdom

**Keywords:** engagement, mental health, mHealth, midwifery, perinatal depression, pregnancy, self-report, well-being, mobile phone

## Abstract

**Background:**

Maternal mental health impacts both parental well-being and childhood development. In the United Kingdom, 15% of women are affected by depression during pregnancy or within 1 year of giving birth. Suicide is a leading cause of perinatal maternal mortality, and it is estimated that >50% of perinatal depression cases go undiagnosed. Mobile technologies are potentially valuable tools for the early recognition of depressive symptoms, but complex design challenges must be addressed to enable their use in public health screening.

**Objective:**

The aim of this study was to explore the issues and challenges surrounding the use of mobile phones for the self-report of psychological well-being during pregnancy.

**Methods:**

This paper presents design research carried out as part of the development of BrightSelf, a mobile app for the self-report of psychological well-being during pregnancy. Design sessions were carried out with 38 participants, including pregnant women, mothers, midwives, and other health professionals. Overall, 19 hours of audio were fully transcribed and used as the basis of thematic analysis.

**Results:**

The study highlighted anxieties concerning the pregnancy journey, challenges surrounding current approaches to the appraisal of well-being in perinatal care, and the midwife-patient relationship. Designers should consider the framing of perinatal mental health technologies, the experience of self-report, supporting self-awareness and disclosure, providing value to users through both self-report and supplementary features, and designing for longitudinal engagement.

**Conclusions:**

This study highlights the needs, motivations, and anxieties of women with respect to technology use in pregnancy and implications for the design of mobile health technologies.

## Introduction

### Background

Perinatal depression (PND) affects up to 15% of women during pregnancy or within 1 year of giving birth in the United Kingdom (UK) [1]. The occurrence of PND may be as high as 35% in certain demographic groups [2] and may affect up to 10% of men [3,4]. Suicide is the leading cause of maternal mortality within a year of birth [5].

Pregnant women suffering from depression are more likely to engage in unhealthy practices, including poor diet, substance abuse, and failure to enroll in prenatal care, and are at increased risk of self-harm and suicide [6,7]. A meta-analysis points to antenatal depression as the strongest predictor of postnatal depression [8]. In addition, antenatal depression can affect fetal development and has been identified as an independent risk factor for children’s behavioral, cognitive, and emotional development through adolescence [7,9-11].

Timely identification of depression and depressive symptoms can therefore enable early intervention, reduce the likelihood of developing postnatal depression, prevent more severe forms of the condition, reduce its intergenerational impact, and improve a woman’s overall health status [5,7]. In the context of the UK’s National Health Service (NHS), mental health assessments in pregnancy are typically carried out verbally and using paper-based questionnaires completed in the waiting rooms of midwifery clinics. The National Institute for Health and Care Excellence guidelines recommend the use of the 2-item Generalized Anxiety Disorder scale (GAD-2) to screen for anxiety in the early stages of pregnancy, followed by further screening using the GAD-7 scale, Edinburgh Postnatal Depression Scale (EPDS), or the Patient Health Questionnaire (PHQ-9) if a risk is identified [12].

However, it is estimated that at least 50% of PND cases go undiagnosed [13,14]. Depression during pregnancy is marked by an unwillingness to seek help at what parents believe should be a happy time and difficulty separating symptoms of mental illness from normal fluctuations in mood [15]. Although almost all UK-based midwives (96%) report asking women about their mental well-being at their first appointment, only 1 in 10 women recall being asked and almost half report never being told about the possibility of mental health problems [16].

### Perinatal Mental Health Technologies

Mobile devices have the potential to facilitate the remote screening and monitoring of mood and depression throughout the antenatal period, extending care to underserved and at-risk populations, enabling timely assessment and intervention, gathering ecologically valid and longitudinal data, overcoming stigma, supporting honest disclosure and fostering trust between women and midwives.

In the perinatal context, previous design research has examined prototype technologies for infant health tracking [17-19], as well as information provision and seeking behaviors, as in the case of Babywijzer, a prototype mobile app for Dutch women [20]; Baby+, a food and weight tracking and health information app for Pakistani expectant mothers [21]; a short message service text message-based system for the personalized communication of health information to pregnant women in Kenya [22]; and qualitative analyses of women’s use of technology for information seeking [23,24].

Other prototype apps have focused on health data tracking. Bloom supports recording of nutrition, hydration, activity, weight, symptoms, and mood [25]. Nuwa aims to facilitate communication between women and their partners [26]. Qualitative analysis has also examined women’s motivations for the use of mobile apps for menstrual tracking [27]. Peyton et al developed a set of design requirements for mobile health (mHealth) interventions to support physically healthy pregnancies in the context of a “pregnancy ecology” comprising physical, emotional, informational, and social support aspects [28]. Building on this work, Prabhakar et al have proposed a “design framework” for maternal support interventions, the Evolving Ecology of Support, entailing support needs, sources, and interventions [29].

However, design research has yet to address many of the public health challenges that pregnancy presents, including the pervasive stigma surrounding mental health, the potentially severe consequences reflected in mental illness, self-harm and suicide rates [5,7], and the clinical constraints that influence technology adoption, given the role midwives and other health professionals play in shaping the experience of pregnancy.

### Self-Report Mechanisms

Whether conducted using the Whooley questions [30], the EPDS [31], or through conversation with a professional, perinatal mental health screening programs are retrospective in nature. Several decades of cognitive psychology and behavioral economics research have revealed striking differences between retrospective and momentary reports of well-being [32-37], and clinical psychology has faced criticism for neglecting “the dynamics of symptoms” [33], given that “variability over time and dynamic patterns of reactivity to the environment are essential features of psychopathological experiences” [38]. Self-report is not a direct pipeline into consciousness [39].

Mobile devices have enabled us to expand our inquiry to real-world contexts through ecological momentary assessment (EMA); the self-report of experience in the moment and over time [40]. However, although design research has examined mobile apps for bipolar disorder [41], depression [42], and anxiety [43], how different reporting mechanisms shape the experience of self-report and the relationship between these perspectives have largely been underexplored to date [40].

### User Engagement

Designing effective self-report technologies in large part hinges upon the engagement of users, often over long periods of time, whether to collect valid data for mental health screening, behavioral change, personal health-related outcomes, or self-awareness [44,45]. Previous studies have found that longitudinal self-reporting tends to swiftly decline following “an initial burst of interest” [46,47]. Understanding users’ motivations for engagement is therefore a key step in the design of mHealth technologies, particularly in the case of those designed for self-report [47,48]. Previous research has examined how users’ engagement with mHealth systems is shaped by the provision of feedback [47], data visualization [49], and multimodal input [50]. However, there remains a need to explore the factors that influence longitudinal user engagement in the perinatal context.

### BrightSelf

We explore these issues in the design of BrightSelf, a mobile app and clinical interface for the self-report of psychological well-being in pregnancy. This system was developed through collaboration between public health and human-computer interaction (HCI) researchers for deployment in a multisite clinical study using a randomized controlled design to examine the capacity of mobile technologies to facilitate the longitudinal, momentary, and retrospective monitoring of antenatal mood and depression [51].

Toward these ends, the primary objective of this study is to obtain insight into the experience of pregnancy and perinatal care, motivations, anxieties and concerns of parents, aims and responsibilities of health professionals, relationships between women and public health services, and the use of technology in pregnancy. This qualitative design research allowed us to identify requirements for a mobile app for deployment within a public health service, explore concept development with users, and perform iterative prototype evaluation.

## Methods

### Approval

This research forms part of an interdisciplinary clinical research study reviewed and approved by the National Research Ethics Service Committee South East Coast. A research ethics submission for this design research protocol was submitted to and approved by the Head of the Department of Primary Care and Public Health and the Joint Research Compliance Office Coordinator at the same institution.

### Design Sessions

This research involved an iterative series of design sessions. Sessions began by exploring design challenges through open discussion, including how technology shapes the self-report of well-being, how users engage and are engaged in the honest disclosure of mental health concerns, how health professionals might act upon reports of psychological well-being, and how technology might contribute to our evolving conception of perinatal well-being and its pursuit, followed by concept development and prototype feedback.

Participants were recruited through social media, the distribution of cards and posters, distant acquaintances, and contact with midwifery clinics. Individual sessions were arranged to support the inclusion of women and other individuals who were unable to attend group sessions or preferred to discuss their experiences independently. Six sessions with women were conducted over Skype at their request. Sessions were conducted by a transdisciplinary and mixed gender team of 1-3 HCI and public health researchers between April 2016 and August 2017 and lasted 1-2 hours. Colocated sessions took place in the London and Cambridge area.

### Participants

Design sessions involved 38 participants. Five group sessions, each with 4-7 participants, were attended by 15 practice and research midwives, 1 clinical studies officer, 1 psychologist, and 4 medical researchers or clinicians in maternal health, obstetrics, and midwifery. In addition, 17 individual sessions were held with 8 pregnant women, 3 mothers, 2 general practitioners (GPs), 1 clinical psychologist, 1 child and adolescent psychiatrist, and 2 maternal and child health researchers. The health professionals who participated in these sessions were aged 25-60 years, had a variety of ethnic backgrounds, and experience working with pregnant women through practice and research.

Table 1 illustrates the demographic characteristics of maternal participants; 7 of these 11 participants had experienced at least 1 miscarriage. Two participants (P7 and P11) had been previously diagnosed with depression, and 1 (P5) with anxiety. However, no participant had received a specific diagnosis of PND. All professed “good” to “excellent” abilities with technology, all were in stable relationships, and all held a university or college degree.

**Table 1:** Demographic characteristics of maternal participants*.*

Abbreviation	Week of pregnancy	Previous pregnancies	Children	Age (years)	Ethnicity	Nationality
P1	27	0	0	28-32	Asian British (Indian)	English
P2	Not pregnant	2	1	≥38	Mixed Other	Singaporean
P3	22	0	0	28-32	White British	English
P4	16	0	0	28-32	White Other	French
P5	11	1	0	≥38	White Other	American
P6	35	1	0	33-37	White British	English
P7	10	0	0	≥38	White Other	Greek
P8	31	3	2	33-37	White British	English
P9	Not pregnant	5	2	33-37	White Other	Lebanese
P10	Not pregnant	4	3	33-37	Mixed Other	Singaporean
P11	39	2	1	33-37	White Irish	English

### Analysis

In total, 19 hours of audio were recorded, transcribed in full, and subjected to thematic analysis. The analysis was conducted in parallel by 2 primary authors working both inductively and deductively with respect to key design challenges and issues arising within sessions related to well-being, perinatal care, self-report, technology adoption, and engagement. This paper highlights the voices of women, introducing clinical perspectives where pertinent. By complementing the directly reported experiences of women with a wide sample of health professionals, we included phronetic input from those with everyday experiences of vulnerable populations, including younger women, ethnic minorities, and women experiencing domestic abuse [52].

## Results

### Themes and Subthemes

Participants described pregnancy as “a bit of a journey” punctuated by both positive and negative experiences, “good days and bad days” (P3). Textbox 1 presents an overview of the themes which emerged from the analysis.

### Pregnancy: A Bit of a Journey

As a pregnant woman you think in weeks, you really do.

P3

Pregnant women described thinking about pregnancy in terms of multiple concurrent timescales, trimesters, months, and weeks. This patterning shapes parents’ reflections and provides context for descriptions of key moments: “Oh I felt my first movement at 17 weeks it was so exciting…it was amazing at 14 weeks when I stopped feeling sick” (P3).

This was described by one participant as a period of transition and expectation during which “the notion of time is essential but it’s always looking forwards” (P4). Yet, comments from other participants suggested that parents’ reflection comprises past, present, and future focus: “Is that the kind of parent I want to be later, and if not, why start now?” (P5).

Pregnancy is a time of change, frequent emotional change, but also change of a more enduring form, to self-identity, and a “shift within the couple’s life stage” (P4). Emotional changes were described as “hormonal roller-coasters” (P8), crying “and the next minute” laughing (P4), which can lead mothers to feel less “emotionally resilient” (P8). This vulnerability can be compounded by a “loss of identity,” “not at all kind of taken up or kind of considered in your normal health care” (P6). Pregnancy, particularly in the first instance, is a significant chapter in parents’ lives, and yet, as one first-time expectant mother recounted, “I don’t want it to be the only thing going on for me” (P1).

### Positive and Negative Experiences

When describing pregnancy, women recounted positive experiences, such as ultrasound scans, “really nice shared experiences” (P5) but also did not refrain from expressing feelings of pervasive worry, “when you’re pregnant you do worry about everything” (P3). In addition, health professionals expressed an awareness that “everybody is worried during pregnancy, everybody’s anxious” (Female GP).

**Themes and subthemes.**

The pregnancy journey

- Positive and negative experiences
- Causes of concern
- Pervasive stigma
- Times of pronounced concern
- Experience in pregnancy

Experience of perinatal care

- Midwives roles
- Mental health screening

- Appraisal based on experience
- Indirect appraisal
- Direct appraisal
- Midwife-patient relationship

Technology use in pregnancy

- Information provision
- Communication with care providers
- Fear and worry

Certain participants described particularly intense and distressing events. A mother who had experienced multiple miscarriages described these events as “shocking” and “very devastating” (P9). Her first miscarriage occurred abroad where she described the absence of a midwife “or any kind of support from family and friends.” Following the subsequent birth of her son she stated, “I know I had postnatal depression in the sense that I was always scared that I might die and who might look after him, or he might die and how would I feel?”

### Women’s Causes of Concern

The causes of worry and, less frequently, sadness described by mothers were diverse. Women expressed concerns related to heightened awareness of their own physical health, “you are so desperate to get [any problem] sorted” (P3), while avoiding “unnecessary interventions, and extra things done” (P5). Health professionals added that “women often say that they don’t feel that they’re doing a good job” and are “very worried that something they’ve done might be a problem” (Female Midwife). These concerns can pervade everyday life.

You have to bend, and you don’t have to bend, and you have to sleep on the right and on the left. All these are worrying because, you know, even when I sleep, I don’t know if I’m harming my baby.

P7

Women named past experiences, “trying for a long time and miscarriages” (P3), as well as awareness of family history “my brother died…and then my mum had a straight of late miscarriages” (P3) as causes of anxiety. In addition, women expressed future concerns, a fear of giving birth, “I’ve been really struck by others, just quite how terrified they are” (P6), and “scary stories of women not sleeping, of women not having time even to wash their hair” (P7) in the postnatal period. P9 recounted in great detail the anxiety she felt when a nuchal translucency screening revealed a risk of Down syndrome in her fifth pregnancy. Worry then can come to form a vicious cycle, following the belief that “if you worry a lot you could actually cause yourself to have problems with the baby” (P3).

Not all sources of worry and sadness recounted were related to pregnancy. P4 was going through a period of grieving and was concerned with the impact this might have, “how much the fetus can feel.” Many midwives pointed to the presence of other children in the home as a common source of stress. Finally, women talked about their relationships with their partners. For one woman (P5), the concern was to avoid disconnection with her husband by keeping him involved in the pregnancy. For another (P4), her husband was eager to participate yet she found that “it’s really difficult to find the place of your partner…a very silly thing, but when you go to midwife there is no male toilets. It’s these kind of things.” These concerns led, for many women, to an urgent “need to know” whether what they were experiencing was “normal.”

### A Pervasive Stigma

Women and health professionals universally described a “massive stigma” surrounding mental health during pregnancy. This was articulated in terms of shame (P9), fear (P8 and P9), pressure, obligation, and guilt (P6); “to report that you’re not happy during pregnancy, you’re just not meant to” (P6), “no-one is afraid to tell their midwife they have pain when they’re pregnant but you are afraid to say ‘well I’ve been having these dreams…’” (P11). This taboo ranged from the need to maintain “a certain image of it…when you sit in clinic” (P5) to a fear of inadequacy as a mother (P4).

A thread was revealed in women’s comments of a society “very quiet” (P6) on many of the less pleasant experiences of pregnancy, “it’s not what you see on the TV” (P6), resulting in false expectations, and a lack of support when problems do emerge, “I was amazed at how many people said ‘that happened to me too’ but I’d never heard people say it before” (P6), leaving women with the sense that they need to deal with concerns alone, “it’s probably hormonal, it’s probably postnatal depression, the fact that I’m always worried” (P9), and subsequently upsetting women’s trust in others.

Just being told to go and get on with it, and not to worry too much because it was completely normal, but the message that it’s completely normal when you’ve never heard about it or seen, kind of, any references to it, is not very convincing.

P6

Stigma was linked to a fear of particular consequences. Foremost among these is the fear that “if I told them too much they might take my baby away” (P11). Among midwives, this fear was frequently discussed.

For them it’s a real threat that you’ll take the baby away from them and I mean I can appreciate where they’re coming from because you get it in the papers “oh they’re taking a baby away.”

Female Midwife

Furthermore, women expressed a fear of being placed on medication, which could cause harm to their child antenatally, “one of the biggest fears I had was that if I told the midwife about those feelings there was a chance they might…put me on a medication that might harm the fetus” (P11), or affect their ability to breastfeed, “if you’ve gotta take whatever drugs the doctor’s gonna give you, that’s gonna go in the breastmilk, and that’s gonna affect your baby’s brain” (P8).

### Times of Pronounced Concern

**Figure 1:**
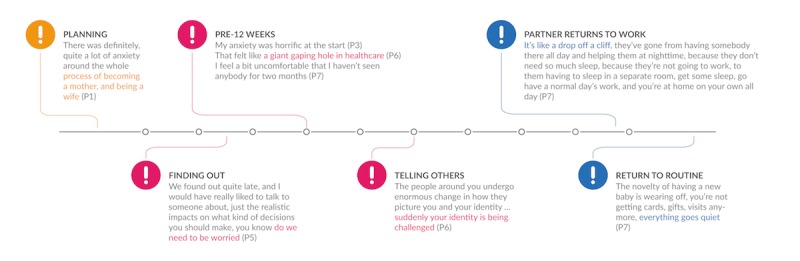
Times of Pronounced Concern.

Descriptions of negative experiences point to gaps in care and opportunities for effective assessment and support. Participants spontaneously described as times of particular anxiety; the planning period (P1), first discovering the pregnancy (P3 and P5), the weeks spent waiting to meet the midwife (P3, P5, P6, P9, and P11), telling others of the pregnancy (P6), the 30^th^ week (P3), and specific weeks postnatally (P8; Figure 1).

In particular, the period between notifying a GP of the pregnancy and the first midwife appointment was described as “really difficult” (P3), “a time of high anxiety” (Female GP) and “very isolating” (P11). These feelings were exacerbated by a lack of support (P6 and P7), morning sickness (P11), bodily changes (P6), the pressure to hide pregnancy (P6), a fear of complications arising from comorbid conditions (P7), and anxiety-induced Web-based searching (P3). Miscarriage often occurs during this period, and women described this as “very difficult from a mental health point of view actually, you are just sort of told ‘Sorry, that was a miscarriage, off you go’” (P6). Recognition of the first trimester as a medical and informational gap in line with changes to women’s needs over time mirrors previous design research findings [23,29].

### Experience in Pregnancy

Striking among participants was the difference that experience in pregnancy made to their outlook. Mothers with multiple children, on the whole, spoke of pregnancy in less emotive terms. Comments made by these women reflect what they had learned and come to expect, “I know, having had two babies myself and expecting my third, that the reality is very much different” (P8).

Experience grants these women knowledge, which helps them cope with difficult circumstances, knowledge concerning “how normal those rollercoasters are and how those terrible crazy dreams are” (P11). This experience applies not only to the emotions of pregnancy but also to the health care system, its pathways, and the likelihood of undesired consequences. These women’s experience is a potential resource for first-time mothers but does not always take that form. P5, an American woman in her 11^th^ week, found that maternity advice could be condescending “you can do pregnancy wrong or you can do it right” and that “as a first-time pregnant lady, everyone’s like ‘oh but don’t you know?’” P6 described feeling “quite rapidly that you’re less competent than you were before.”

### The Character and Experience of Perinatal Care

**Figure 2:**
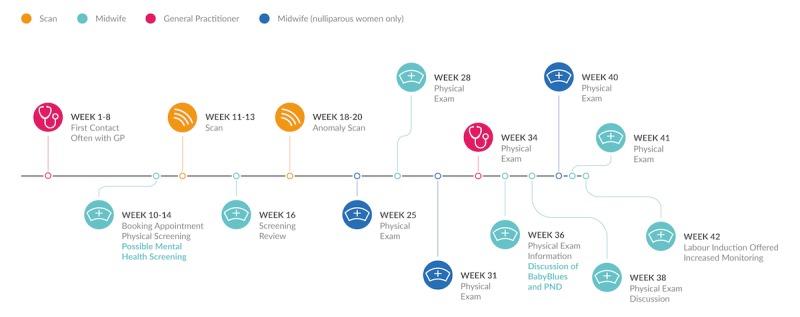
The antenatal care pathway. PND: perinatal depression.

The majority of NHS antenatal care is performed by midwives (Figure 2). A woman’s first meeting with her midwife, termed the booking appointment, usually takes place within the first 12 weeks of pregnancy; this appointment is typically scheduled following an initial visit to a primary care provider, such as a GP, and can last up to 2 hours. Between 8 and 12 meetings usually follow between a woman and her midwife. The number of appointments and scans provided varies depending upon the risk to the mother and child, and local resources. In certain settings, there is a discontinuity of care, with some women seeing a different midwife at each subsequent appointment. P6 commented that as a result the very notion of “your midwife” proves confusing early in pregnancy.

### Midwives’ Roles

Midwives referred to their goals as ensuring the well-being of the mother and baby, monitoring the mother’s vital signs, examining the mother’s past medical history (including mental health), performing a risk assessment, and if necessary making referrals, coordinating care and covering social needs, creating a care plan, reassuring women and their families, providing information, signposting what choices and resources are available, establishing trust and forming a relationship.

### Mental Health Screening

#### Midwives’ Views

Midwives do not see themselves as mental health professionals, as was reflected in discussion concerning the use of the EPDS in clinic; for example, “*we’re not qualified to diagnose so it is then up to us to pinpoint the appropriate professional to direct them to* ” (Female Midwife). Midwives are also intensely aware of the treatment gap that exists with respect to PND, as illustrated by the following:

Everyone that comes through the labour ward, it’s depression, depression, depression…especially the ones that have lost their babies and I didn’t have time to speak to one of them...two days later she committed suicide…So I know there is a gap, definitely.

Female Midwife

Women recounted a marked variance in midwives’ approach to mental health, a complex and nuanced form of interaction.

#### Appraising Well-Being Based on Appearance

Several women stated that their mental health and mood was rarely discussed, “I don’t know if I had a special case where the midwife generally doesn’t ask anything about your mental health or stress or worries or concerns” (P9), “I don’t think there’s been anything, anything that’s been put in front of me during my pregnancy that has focused on mental health” (P6). A female GP described how “sometimes people don’t want to pick up problems that might take more time” and employ “leading comments like ‘Well you look like you’re fine don’t you?…Things are going really well…you look great!’” This line of thought echoes the comments of a community midwife participating in previous research, “The services are not there to support women and why open a can of worms that you can do nothing about” [16].

#### Appraising Well-Being Indirectly

Women also described a conversational approach employed by some midwives, “they might ask you how you’re feeling but that’s a really general question isn’t it?” (P8). This light-touch approach is often motivated by midwives’ appreciation of the stigma surrounding mental health. It is therefore worth noting that women described a clear awareness of midwives’ intentions, “I know what they’re asking.…” (P10), “that is the question that they were trying to ask” (P9).

#### Appraising Well-Being Directly

Another approach recounted by women involves direct questioning during the booking appointment, including the verbal application of the Whooley questions. Some women found this approach “so weird…I had no idea what I was doing being pregnant” (P6). Two women (P2 and P3) compared this to a routine question about domestic abuse, which also took them by surprise. Some midwives also expressed doubts whether this approach does “probe as deeply as perhaps is needed sometimes.”

### The Midwife-Patient Relationship

The midwife-patient relationship is a primary feature of antenatal care and a key factor in the success of mental health screening. Several women described midwives as “very supportive and nice” (P9), “amazing” (P8), and “very lovely people” (P10) who “care really deeply” (P11). Women’s perceptions of midwives’ roles coincided for the most part with midwives’ descriptions, there “to aid you through” (P3).

In addition, 2 women expressed the sentiment that “a midwife is not a mental health professional” (P11), “it’s not that I would have wanted her to [ask me about my mental health], I never expected her to, I never thought that was something they actually covered” (P9). Instead, midwives were described as generalists, a disappointment for P9, “all the reasons for nuchal translucency…even when I spoke to the midwife, I couldn’t really talk to her about these things.”

Midwife appointments were described by some women as “very routine,” “more concerned about taking measurements” (P2), “you go in, and you do the samples, and you do the tests, and it’s like ‘see you next time.’” For P4, a woman in the 16^th^ week of her first pregnancy, this was not a positive interaction, “after my first meeting with my midwife I was very upset about this…look into my wellbeing, I’m a mother, and I’m not just a number, I’m not just…a blood test.” Women also felt that their initial classification as low or high risk dictated subsequent interactions with their midwife. Women classified as low risk (P1 and P4) were critical of this approach, “low-risk, from a medical perspective is not the same as well-being, and you still have questions” (P4).

NHS midwives described significant time pressures in their work, and women expressed awareness of this, often in sympathetic terms (P9, P6, and P11). However, this pressure also impacted women’s willingness to disclose concerns, “they basically just kick off my appointments with ‘Oh great, you’re entirely normal, this is gonna be really quick’” (P6), “I did have questions for her but I just felt like she was in a rush” (P1).

### Technology Use in Pregnancy

Women frequently described technology as playing a role in their pregnancy. All owned mobile phones and used websites related to pregnancy. Six participants owned tablet computers and only one had not used any mobile app for pregnancy (P7). Many women used email (P8 and P9) and phone (P1, P3, and P8) to contact their care providers. P9 remained in touch with her UK-based gynecologist by email while traveling abroad. A well-timed notification from an app helped P3 to identify a water infection. P6 and P1 described using app content as a way to engage their partners in their pregnancy, “a sort of third party thing that you can point at and say ‘Look, this is what might be coming up next’.” Women most frequently described using technology to seek information, particularly to overcome perceived shortcomings in care (P3 and P9), a behavior portrayed by women participating in previous research as a means to compensate for “useless” and “overwhelming” printed literature [24].

However, the use of technology was not always recounted positively. P6 found herself targeted with Web-based advertising related to her pregnancy, “Facebook notices you’re pregnant…Google also knows I’m pregnant now.” Searching for information over the Web could be overwhelming, “a pit of anxiety,” a “terrifying free-for-all” (P6), and the removal of international barriers did not always help, “you can read things that just make you more anxious because you don’t know why you’re not getting that care” (P6).

## Discussion

### Design Implications

In this section, we discuss the design implications of this study for BrightSelf and apps for perinatal psychological assessment in general (Textbox 2).

In its final form, BrightSelf, a mobile app (Android and iOS) designed for deployment within a public health service, supports the self-report of well-being in pregnancy and provides a number of supplementary features, including an interactive visualization of users’ data, concise well-being and support information, and an *ideas machine*, which dispenses animated well-being tips (see Multimedia Appendix 1). Momentary reports are collected using visual analog scales for mood, sleep, worry, enjoyment, and energy, as well as 2 contextual questions concerning semantic location and activity, constructs chosen according to participants’ comments and prior research [53]. In compliance with existing NHS care pathways and the National Institute for Health and Care Excellence guidelines, retrospective reports take the form of the EPDS, a self-administered 10-item survey that assesses feelings of guilt, sleep disturbance, anhedonia, and suicidal ideation present during the past 7 days [31]. Users are reminded to provide reports over time using notifications.

### Importance of Appropriate Framing

How do people position it? How do you want to position yourself?

P4

These design sessions highlighted women’s intense need to understand their own well-being in pregnancy and the challenges faced by health professionals attempting to do so. Tension between these two perspectives necessitates the careful framing of any technology introduced into the perinatal context.

#### Aversion to Mental Health

A lot of people, as soon as you say the word mental health shut up, completely.

P4

Women described themselves as low, anxious, and in need of support, and yet were keen to distance themselves from any labels connected to mental health or illness. This intense disconnect with the term “mental health” suggests that employing this language can reduce the reach of mHealth technologies [42]. We asked women about their thoughts concerning other terms such as “psychological well-being.” While some preferred this term to mental health, others felt it possessed similar negative connotations, “psychologist, psychiatrist, mental…for me, sit in a similar category” (P8). Several women spontaneously proposed “emotional well-being” as preferable.

#### Introducing an App

The initial booking appointment provides an opportunity to scaffold the introduction of an app to women. Both women and professionals stressed the need for this first impression to be a normalizing experience, “same as you go to see [the] midwife for the booking appointment, it’s just something that everyone does” (Female Psychologist).

This interaction illustrates how the pathways of clinical care constrain technology design in the perinatal context. Although women highlighted the weeks before the first appointment with a midwife as a time of high anxiety, there is currently no opportunity for midwives to interact with patients in this period, and even if expectant mothers were provided with a screening tool, no pathway exists to support subsequent action.

#### Every Pregnancy is Unique

Pregnant women are a highly diverse demographic. Midwives also demonstrate marked variance with respect to their experience and practice. In the case of mental health screening in particular, it is important to avoid design choices that might imply pregnancy planning, the presence of a supportive partner, family relationships or history, a lack of complications in pregnancy, infant development, the presence of other children, or the occurrence of miscarriage.

### Supporting Value in Self-Report

I’m not going to just use it because you ask me to use it, right.

P10

Women emphasized that they would not comply with the burden of self-report without recognizing value in doing so. While some participants expressed reservations that engaging in self-report might reinforce negativity, lead to obsession or a biased perception of their own well-being, others reported motivations related to understanding their subjective experience, to relationships with others and to possible actions.

#### Turning Inward

Women expressed value in the potential of self-report to enable and maintain self-knowledge, progress, and well-being-related change. Less anticipated were those motivations that related simply to supporting self-awareness and reflection—comparing trimesters, checking in “treating it like a game” (P5), venting “to someone or something” (P9), acknowledging emotion, and preserving experience.

#### Turning Outward

Other motivations for the use of self-report technologies reveal the socially situated reality of pregnancy. Women envisioned the use of data to both avoid feeling alone, “the reason why everyone Googles so much” (P3), and to feel normal, “Is this normal? Am I normal?” (P10). Health professionals often spoke of women’s need to know what’s normal, an important factor in the success and failure of mental health screening programs, “they know they’re struggling, but they’re not sure if everybody, if that’s normal” (Female Psychologist).

#### Taking Action

Finally, women described how the potential of self-report data to support action could also motivate its collection—inciting conversation “to ask the right questions” (P4), inspiring action “I’m suffering from something, I should do something about it” (P10), obtaining support, and enabling midwives to tailor care “to really move away from high and low-risk pregnancy” (P4).

### Shaping a Better Self-Report Experience

Self-reports of well-being are colored by the experience of reporting. The methodological concern of reactivity, for example, whether we are “tapping a phenomenon as it exists, or as it has been transformed by measurement” [54], translates into a design constraint for self-report systems.

**Figure 3:**
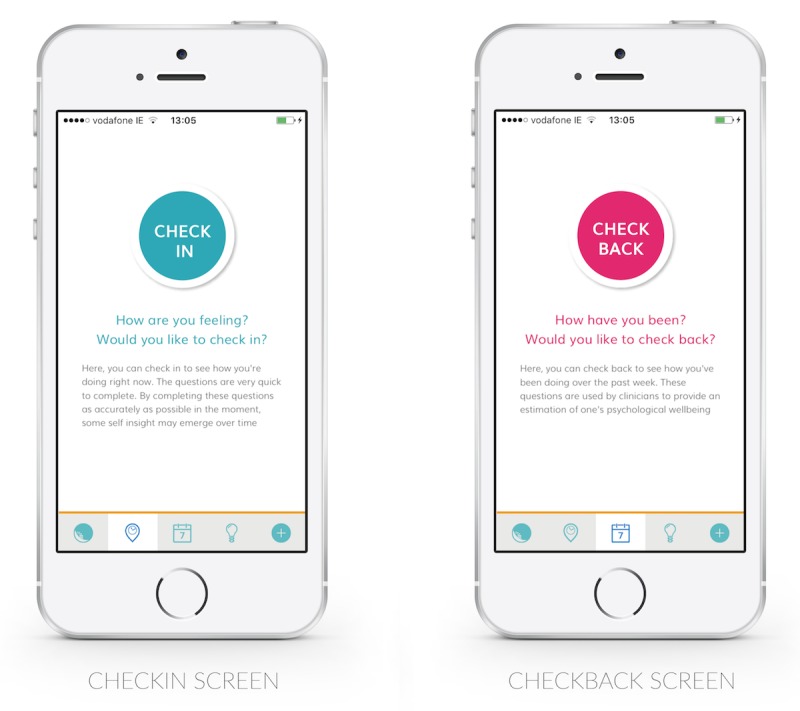
Check In and Check Back screens.

Participants in these sessions expressed strikingly different reactions to the experience of retrospective (EPDS) and momentary (EMA) reporting. The medical tone of the EPDS often immediately evoked guarded reactions among women, “see, this is all the mental health stuff…” (P5), “now I‘m being like ‘Ooo I’m being evaluated so I need to be careful’” (P4). Certain questions evoked particular criticism. Question 4, which asks whether a woman has been “anxious or worried for no good reason,” led participants to exclaim “what if you’re anxious for a good reason” (P5), “women hate it!” (Female Psychologist). Questions regarding “sad or miserable” feelings and crying were also described as difficult to complete when pregnant. Terms like *EMA*, *momentary*, *retrospective*, and *assessment* reflect medical and academic framings. We identified the terms “Check In” and “Check Back,” which were perceived more positively by users (Figure 3).

**Figure 4:**
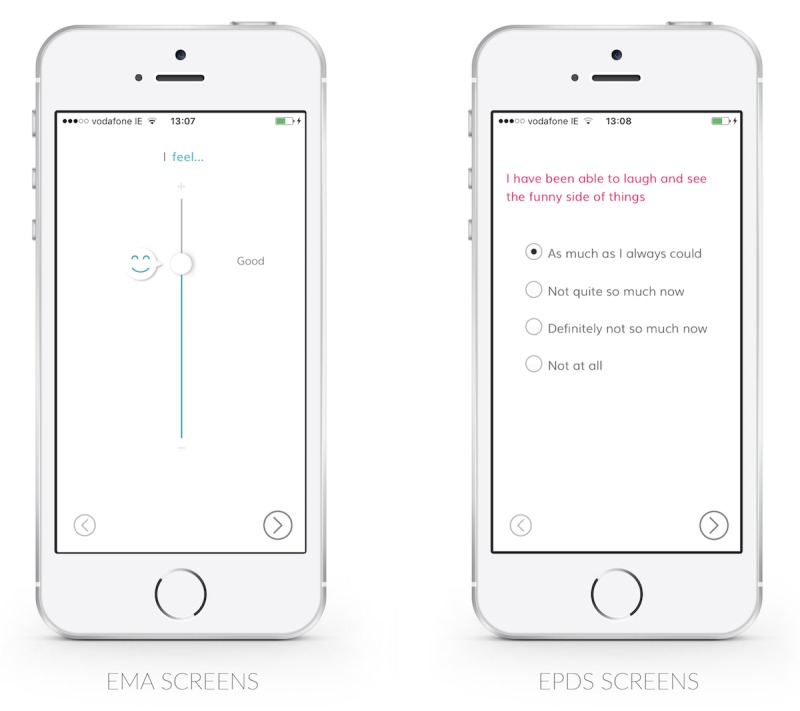
Screens showing ecological momentary assessment (EMA) and the Edinburgh Postnatal Depression Scale (EPDS).

A proposed EMA reporting experience, in contrast, was described as “really easy and not intrusive” (P6), “light-hearted” (P3), and “really nice and almost a bit fun as well” (P1). In comparison to the EPDS, these momentary measures reflect a broader spectrum of well-being, including positive dimensions. This may allow women to present a more complete appraisal of their well-being and to see reporting less as “a test that you pass or fail…a box tick exercise” as one female GP described the EPDS. In addition, the use of graphics and animation can make EMA a more engaging interactive experience (Figure 4). Women reported that they would complete EMA reports more often and with greater honesty.

**Design implications.**

Framing

- Avoiding “mental health”
- Introducing an app
- Uniqueness of each pregnancy

Supporting value

- Turning inward
- Turning outward
- Taking action

Shaping self-report experience

- Being evaluated
- Less intrusive

Self-awareness and self-disclosure

- A light touch
- A safe space
- Empowerment

Designing for Engagement

- Interaction over time
- More than self-report

### Focus on Self-Awareness and Disclosure

Disclosing a need for mental health support during pregnancy requires women to recognize that their symptoms are unusual, accept the possibility of illness, and trust the individuals and services they would approach for support [16].

#### A Light Touch

Peyton et al have encouraged designers of perinatal mHealth technologies to “use immediate needs as a hook for long-term concerns” [28]. This advice was supported by women’s observations that mental health content and assessments should be “coupled” (P9), “couched,” and “sandwiched” (P11) with other features. Women suggested that a lighter touch was key to inviting honest disclosure, “a tool to sort of mention that people often, kind of are, feel differently during pregnancy and that that’s entirely normal” (P6).

#### A Safe Space

Several participants made comments suggesting that technology might make it easier for women to disclose how they are feeling, “through an app I might be a bit more open” (P1), “a safe space where you can articulate some of the anxieties that you’re feeling” (P6). Transparent communication of the potential consequences of disclosure is key to the creation of a safe space [55]. As P11 states, “I think the reality is you know someone’s not going to take your baby away if you tell them that you’re feeling this way but you don’t know whether it means a midwife’s going to come to your house every other day…when you’re in a very anxious moment or in a low period you can’t rationalize.” The interpretation of open-ended data presents clinical and ethical challenges. Women spoke of a desire to provide sufficient context to preserve the meaning in their reported data, “I would want to write the word ‘scan’ under that, or ‘hypo’ under the ones where I’m worried, anxious” (P5). One approach is to include multichoice questions, which allow users to provide a degree of context.

Previous design research has emphasized the need for health tracking apps to provide feedback to users [23]. However, midwives cautioned against labeling mothers’ subjective experience during feedback, “they might think ‘well hold on a minute, I don’t feel that’” (Female Midwife), echoing warnings that technologies should avoid appearing to tell people what to feel [56]. In particular, there was a reluctance among some midwives to present the score generated by the EPDS to users, owing to a concern that it might lead women to “try to beat their score” (Female Midwife), and a sentiment that it would be more valuable to offer an action to patients “you might be feeling a bit low and you might need to speak to a friend” (Female Midwife). Furthermore, women questioned whether increased familiarity with the nature of a screening scale might lead users to “feel pressured to be in that range” (P11) or to provide scores which “they know will get results” (P5).

#### Promoting Responsibility, Action, and Empowerment

In the clinical context, asking questions creates an ethical responsibility to act upon the responses provided [52]; this requires clinical pathways to support appropriate action and can create an additional workload for midwives as well as tensions and inefficiencies in the shared responsibility of a patient’s illness [57]. One midwife indicated that self-report technologies were most likely to be adopted if responsibility was shared across the clinical team.

If it’s a tool to elicit [women’s] true feelings, then that’s only gonna be good isn’t it…but we have to adapt and it would take a bit of planning…for one midwife I think it would be difficult, but if there were a team doing it, that might ameliorate worries.

Female Midwife

Midwives were keen to stress that technology should not act to disempower patients: “it’s about empowering women to take responsibility for their mood and contacting us, rather than me going oh my god I’ve got to bring in all these women because their mood is very low.” It is often reported that mental health professionals experience “much more difficulty in interacting with participants without any progress data” [58], and “difficulty learning from their clinical experiences…when they do not receive accurate feedback” or “when their cognitive processes are inadequate (ie, when they remember information incorrectly)” [59]. Access to women’s self-report data might therefore also serve to empower midwives by providing a more accurate picture of their patients’ well-being.

### Actively Designing for Engagement

Given the burden of reporting, studies employing self-report technologies often report high rates of attrition [48,60]. Women described a variety of features that motivated their use of technology in pregnancy—an appropriate tone, broad appeal, convenience and timeliness, a focus on women’s needs, and anonymity. Women were particularly enthusiastic about a feature of several popular apps that compares the size of the fetus each week to a fruit or vegetable. This provides a visual analogy, allows women to track their progress, and also enables women to raise the topic of their pregnancy in conversation with others.

#### Interaction Over Time

Pregnancy implies a longitudinal perspective. It is therefore essential to consider how interaction unfolds over time. Women’s perceptions of appropriate reporting patterns varied significantly according to the nature of the scale and their concurrent state. Several women felt that reporting 3 times a day for several consecutive days would often be too much, whereas others felt it would prove feasible, for example. Women’s spontaneous insights point toward strategies to support interaction over time, including positive perceptions of mobile notifications, pairing reporting with routine activities, such as Kegel exercises, and the impression that reporting could “counterintuitively” be more rewarding the more it is used.

**Figure 5:**
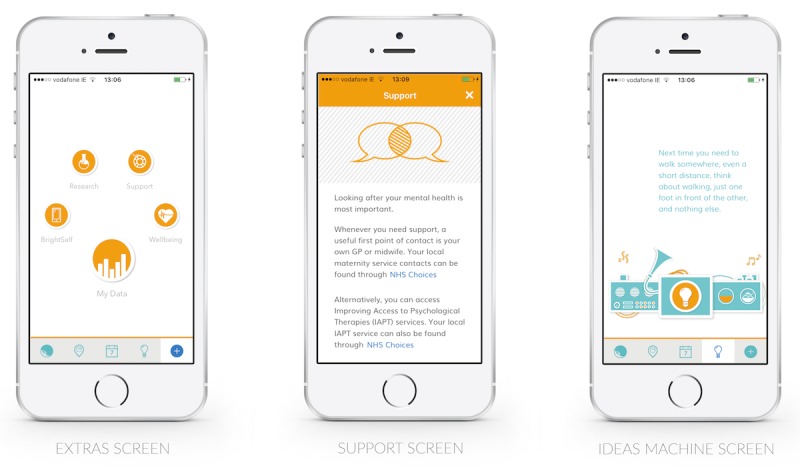
Supplementary features.

Designers can strive to match women’s perceptions of pregnancy, including displaying the individual week of pregnancy and providing regular updates. The timeline of perinatal care (Figure 2) and women’s coincident narratives (Figure 1) point to opportunities for maintaining engagement over time.

#### Value Beyond Self-Report

High rates of dropout and attrition indicate that it is necessary to design for value beyond the act of self-report itself to offset the burden of reporting [48,60]. BrightSelf includes an *ideas machine*, which dispenses microinterventions informed by previous patient-focused research [53] and women’s insights (Figure 5). This feature received highly positive comments from women and was cited as a reason to return to the app. Midwives also supported this feature, but warned against including content that made assumptions about or demanded too much of patients. Such features can serve to lighten the tone of an app, supporting honest disclosure and key clinical needs.

### Conclusions

Appropriate design of engaging tools for the self-report of psychological well-being has the potential to enable greater understanding of well-being in pregnancy, effective mental health screening programs, and the timely identification of depression and depressive symptoms, making treatment and support available to the women who need it. Working with women, midwives, clinical psychologists, psychiatrists, GPs, and other health professionals, this study illustrates how designers can act to support these aims by appropriately framing mHealth technologies, shaping the experience of self-report, supporting self-awareness and disclosure, providing value to users through self-report and supplementary features, and actively designing for engagement over time.

## Appendix

Multimedia Appendix 1 Screen flows, sketches and design rationale.

## Abbreviations

EMA: ecological momentary assessment
EPDS: Edinburgh Postnatal Depression Scale
GP: general practitioner
mHealth: mobile health
NHS: National Health Service
PND: perinatal depression
