# Supplementary material for: A Mobile App for the Self-Report of Psychological Well-Being During Pregnancy (BrightSelf): Qualitative Design Study
Source: JMIR Ment Health. 2018 Nov 27;5(4):e10007. doi: 10.2196/10007 (PMC6290271; doi:10.2196/10007)
Supplement: Multimedia Appendix 1 [file mental_v5i4e10007_app1.pdf]

# BrightSelf

## Sketching
## Screen Flow
## Design Rationale

# Sketching

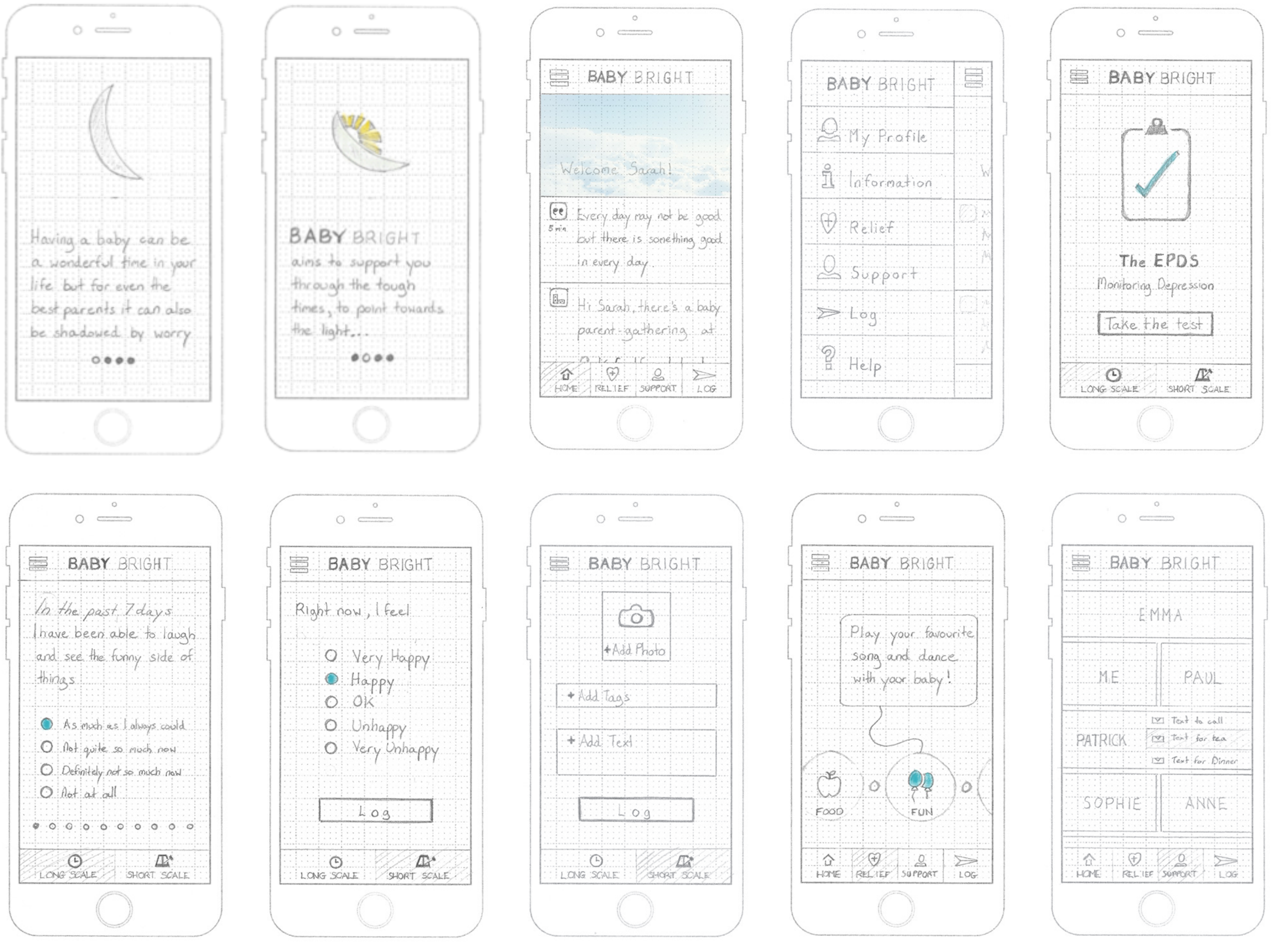
Having a baby can be a wonderful time in your life but for even the best parents it can also be shadowed by worry
BABY BRIGHT aims to support you through the tough times, to point towards the light...
BABY BRIGHT
Welcome Sarah!
Every day may not be good but there is something good in every day
Hi Sarah, there's a baby parent gathering at
HOME
RELIEF
SUPPORT
LOG
My Profile
Information
Relief
Support
Log
Help
The EPDS
Monitoring Depression
Take the test
LONG SCALE
SHORT SCALE
In the past 7 days
I have been able to laugh and see the funny side of things
As much as I always could
Not quite so much now
Definitely not so much now
Not at all
Right now, I feel
Very Happy
Happy
OK
Unhappy
Very Unhappy
Log
+ Add Photo
+ Add Tags
+ Add Text
Play your favourite song and dance with your baby!
FOOD
FUN
EMMA
ME
PAUL
PATRICK
Text to call
Text for tea
Text for Dinner
SOPHIE
ANNE

# Screen Flow

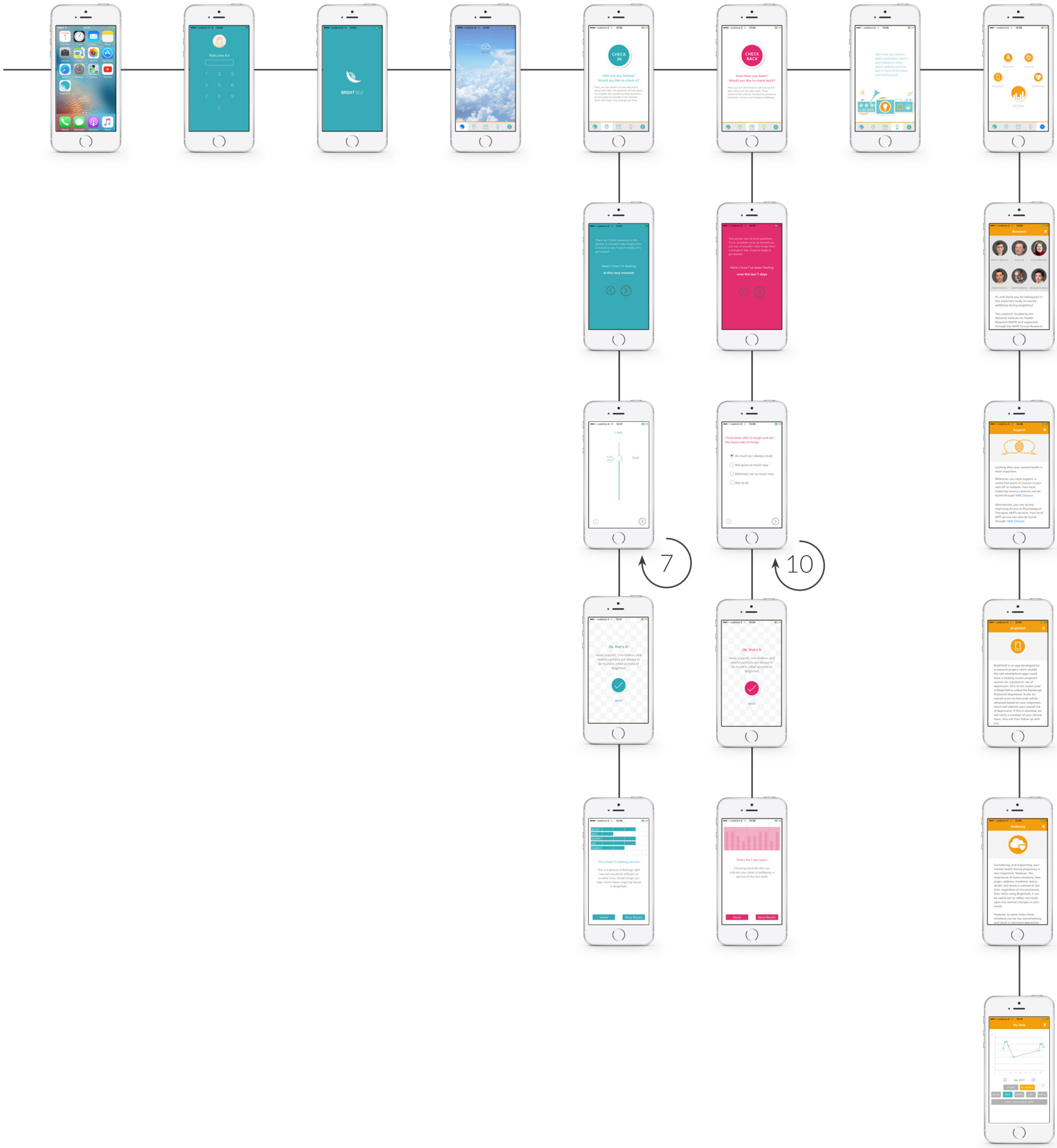

# Design Rationale

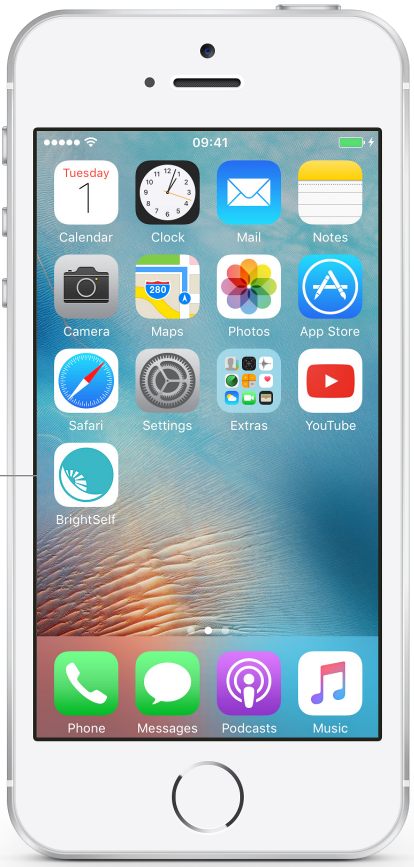


APP LAUNCH

Bright, clean, nonmedical app icon

*"when you say the word 'mental' or 'mental health services', or anything with the word 'mental' it has a negative sound to me" (P8)*

## Colour Scheme

A complementary colour scheme of greens, orange, red and blue was chosen to invite trust and engagement while avoiding a stereotypical pink and flowery styling (See Peyton et al. 2014)

## Baby sling

The constituent shapes of the logo form a Gestalt representation of a baby in a sling,
The appearance of the logo is animated on app launch, a flow designed to suggest the meaning behind each component part.

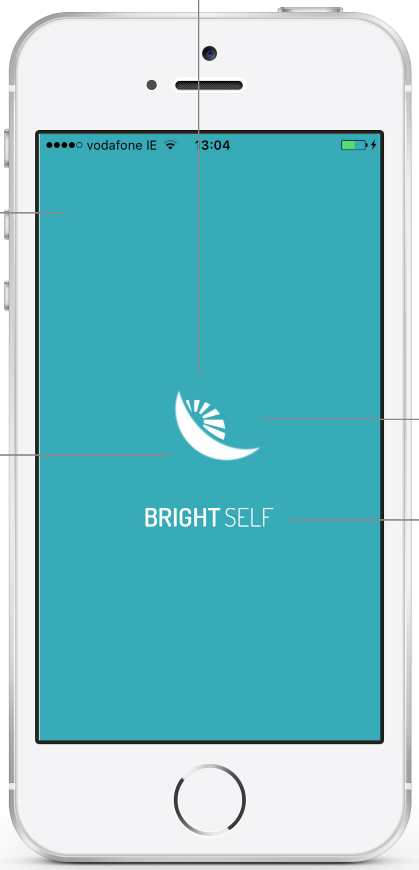


SPLASHSCREEN

## A Moon

The moon represents the opposite half of the spectrum of emotion; night as to day

## Splashscreen

Apple guidelines do not recommend the use of splashscreens, and yet many popular apps maintain their use
To give the app a professional and familiar feel, and to suggest that the user may find value in the use of the app beyond the collection of data to inform research, we chose to include one

## Sun Rays

Bright rays represent hope and positivity

## BrightSelf

Dosis, a friendly, rounded font was chosen to mitigate medical perceptions of the app
A dual weight typeface accentuates the twin meaning in the logo

Profile Icon

A flat icon adds some aesthetic interest to the screen

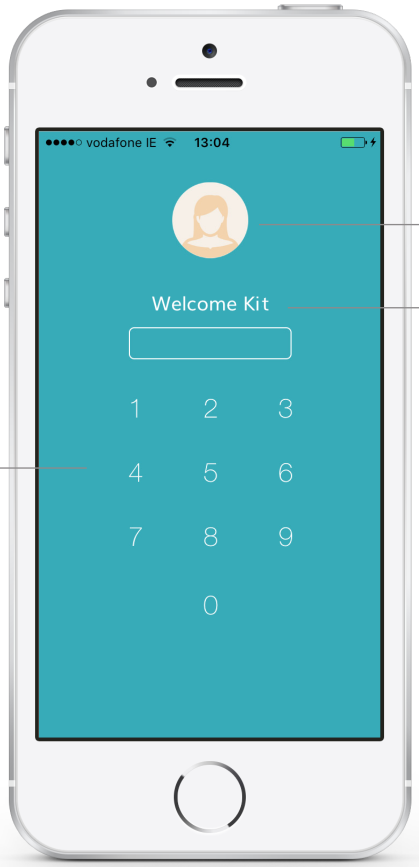


Username

The user's first name is included to foster trust and suggest a personal space

PIN SCREEN

PinCode

A 4-digit pin provides a sense of security
Large numbers allow for quick and single-handed entry, for distracted users

## Syncing status

Icon reflects the now common association of 'cloud storage'
Placed prominently to remind users of the need to sync data, and also to imply the priority of this action
Text confirms the meaning of the icon

## Status bar

A transparent status bar was chosen to support 'presence' within the app

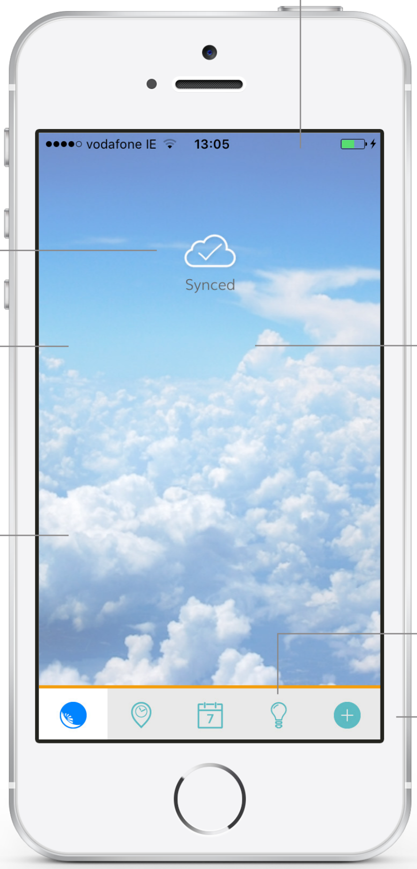


HOME SCREEN

## Feedback Version

A third version of the app presents a scrolling feed on this screen which can display feedback from midwives

## Navigation Icons

Icons were designed to convey meaning at a small scale
Two solid colour circular icons provide symmetry and distinguish the 'Home' and 'Extras' sections

## Initial prototypes included 'inspirational' quotes here

Several midwives deemed this patronising and it was removed

## Single Navigation Bar

A one-handed and distracted user was assumed
This enforced simple navigation
The current tab is highlighted to convey the user's location within the app
An orange horizontal highlight distinguishes the navigation bar from the background

## Nature photographs change each time the app is relaunched

A changing background image provides a degree of novelty which may support engagement

## CheckIn Button

A simple, centrally-located button provides immediate feedback to the user about the purpose of the screen and the action to take. A drop-shadow invites interaction

## Background

A plain white background maintains the user's focus on the purpose of this screen

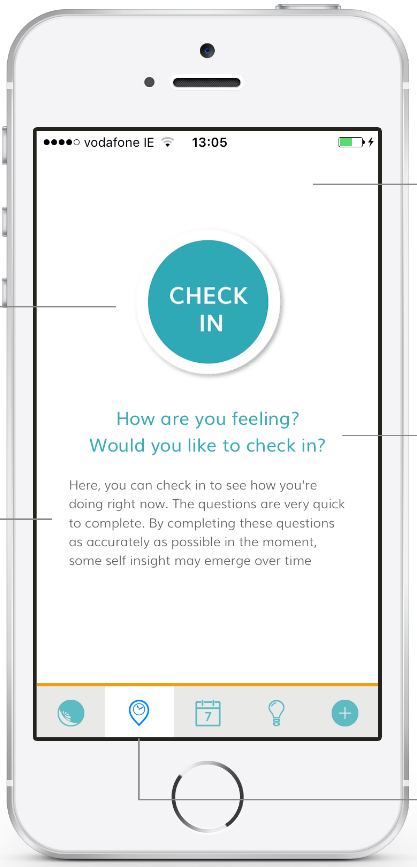


## Subtitle

A subtitle in question form further conveys the purpose of this section of the app

## CheckIn Icon

A location pin emphasises the pinpoint nature of this form of report in time

CHECKIN SCREEN

## Detail Text

Smaller text provides an additional rationale for the use of this mode of report

## Instructional Text

Brief text indicates how long the section will take to complete, important for the engagement of users

*"I think at the beginning stating how long people can expect to do it [to complete a scale] is useful" (Female Midwife)*

## Background

A solid colour background distinguishes this screen from the previous, and demarcates this as the start of a separate section of the app

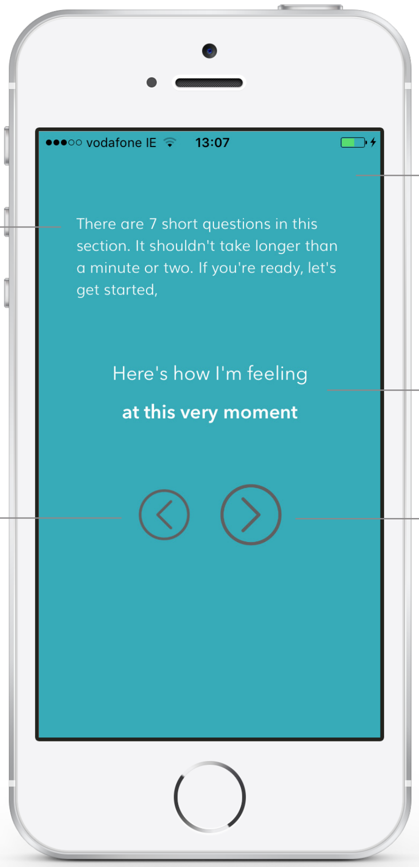


CHECKIN INTRO SCREEN

## Reflective Question

A first-person phrasing indicates the personal nature of this reflection and hints at an individual value in doing so
Bold text emphasises the present nature of these assessments

*"I think 'I' is better than 'You' because it makes the person think that they're actually talking to themselves as opposed to talking to an external thing ... I would be more honest with 'I' than 'You'." (P9)*

## Previous Button

The user is given the option to leave this section, respecting their autonomy, one of three primary psychological needs according to Self Determination Theory (See Ryan & Deci 2008)

## Next Button

A slightly larger arrow icon suggest the way forward
The circular outline invites interaction, a theme maintained through the app

## Question Text

First-person oriented text guides the user's response

## Scale Icons

Flat icons provide a graphical representation of each point in the Likert scale

Icons are animated, increasing in size when the slider is changed and bouncing back when released, supporting engaging and intuitive interaction

## Anchor points

Provide additional feedback regarding the valence of the scale

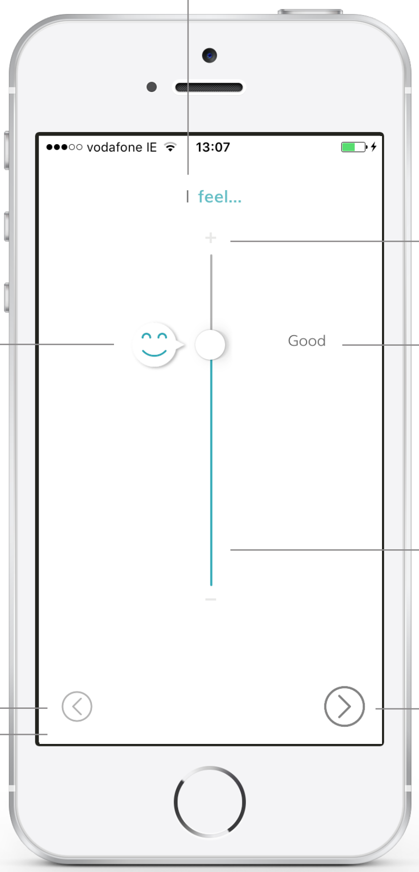


## Scale Labels

Text labels also indicate the meaning of each scale point

## Previous Button

Tracking revisited questions can provide insight into users' patterns of reflection, potential usability concerns and reactivity to reporting

## Slider Track

A minimally aesthetic scale supports sufficient interaction and one-handed use

A change in colour based on the position of the thumb further indicates scale valence

## Background

A simple white background supports efficient interaction, and mitigates distraction

EMA SCREENS

## Next Button

A slightly larger icon suggests progression

## EMA Questions

5 Visual Analogue Scales (Mood, Worry, Rest, Energy, Enjoyment)

2 Radio Button Scales (Semantic Location, Activity)

## Visualisation

An animated horizontal bar chart relays to the user a visualisation of the results they just provided
Simple feedback chosen to support engagement
A minimalist aesthetic is maintained

## Anchor points

Valenced anchor points were chosen over numbers to indicate a wellbeing continuum

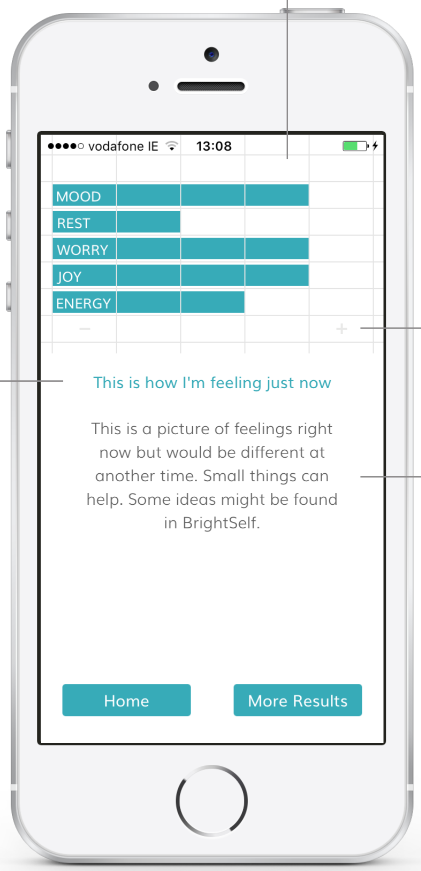


## Text feedback

A single line of feedback in first-person places the results in context
This text is semi-randomly chosen from a set of multiple possible responses based on a sum of a user's reports to provide a degree of novelty and continued incentive

## Detail text

More detailed text suggests actions to take in the case of highly negative results, and promotes self-awareness otherwise
This text is semi-randomly chosen from a set of multiple possible responses based on a sum of a user's reports

EMA RESULTS SCREEN

## Results

Immediate feedback provides value and incentive

## CheckBack Button

A simple, centrally-located button provides immediate feedback to the user about the purpose of the screen and the action to take.
A simple drop-shadow invites interaction
A vibrant red theme distinguishes this section of the app from the Check In section

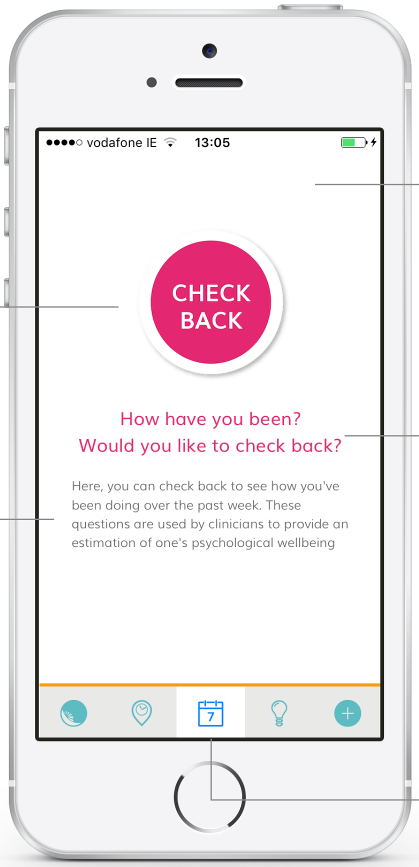


CHECKBACK SCREEN

## Background

A plain white background maintains the user's focus on the purpose of this screen

## Subtitle

A subtitle in question form further conveys the purpose of this section of the app

## Checkback Icon

A calendar icon with the number 7 emphasises the retrospective nature of this form of report

## Detail Text

Smaller text provides a further rationale for the use of this mode of report

## Background

A solid colour background distinguishes this screen from the previous, and demarcates this as the start of a separate section of the app
A vibrant red theme distinguishes this section of the app from the EMA section

## Instructional Text

Brief text indicates how long the section will take to complete, important for the engagement of users

## Reflective Question

A first-person phrasing indicates the personal nature of this reflection and hints at an individual value in doing so
Bold text emphasises the retrospective nature of these assessments

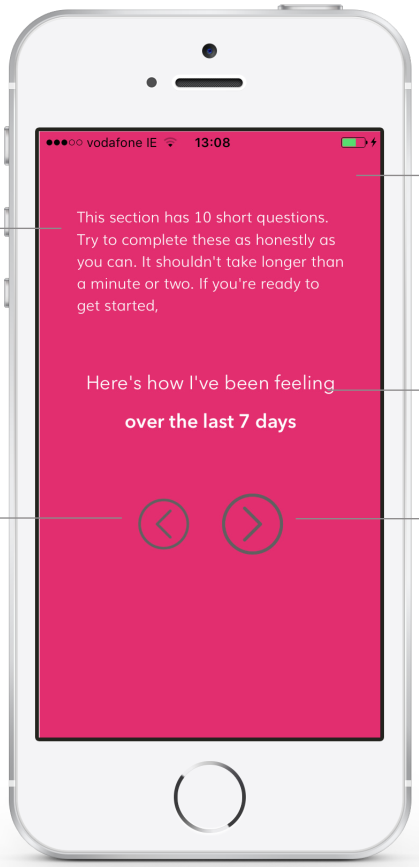


## Next Button

A slightly larger arrow icon suggest the way forward
The circular outline invites interaction, a theme maintained through the app

## Previous Button

The user is given the option to leave this section, respecting their autonomy

CHECKBACK INTRO SCREEN

Question

Red text draws the eye to each question

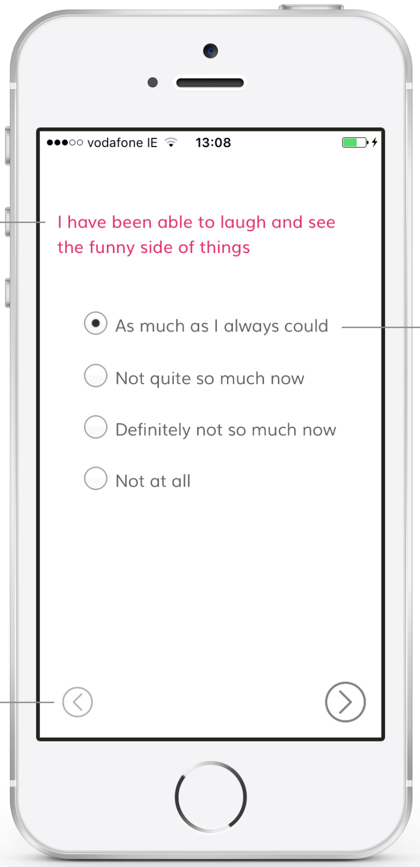


Answer

Radio buttons allow for efficient interaction
The use of circular elements to invite interaction is continued

Navigation

Users can go back and forth between questions

EPDS SCREENS

EPDS Questions

A 10-item scale for perinatal depression screening

## Anchor points

General (intepretivist) rather than numerical (positivist) scoring

## Text feedback

A brief description of the results

## Visualisation

An animated visualisation shows the user’s response to each question, overlayed upon a summative score The choice of whether to display numerical results and their meaning featured strongly in discussion with patients and midwives

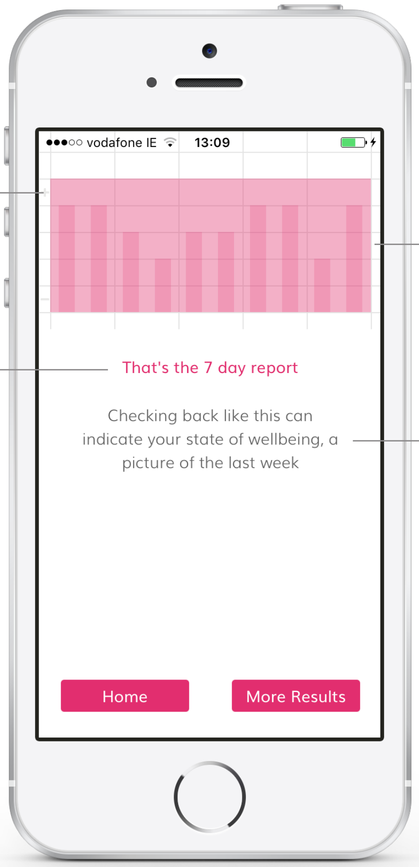


EPDS RESULTS SCREEN

## Detail text

Additional feedback chosen semi-randomly based upon the user’s EPDS score

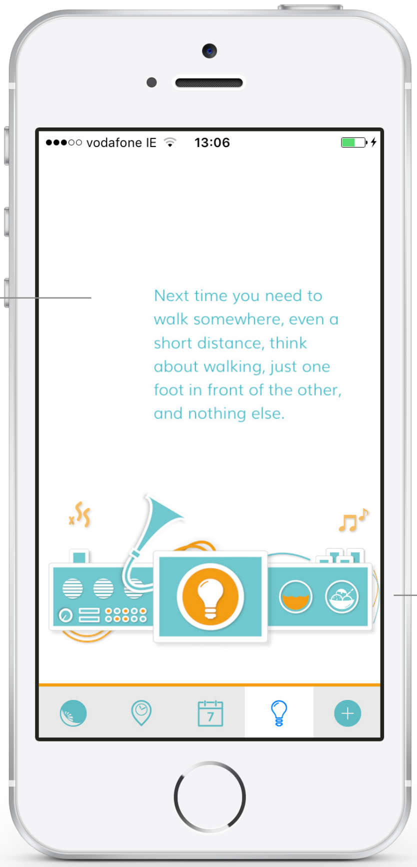


## Idea Text

One of more than 65 tips and activities chosen to support self-care.
The semi-random and numerous selection supports continued use
The light-hearted presentation attempts to avoid a medicalised or obsessive focus

## Ideas Machine

A crate with a lighbulb icon, when clicked expands through a colourful animation into a machine which spouts short sections of randomly coloured text which then float up the screen
The mechanism of interaction (click-based, semi-random, and delayed through animation) was designed to support engagement

IDEAS MACHINE SCREEN

## Additional features

The purpose of this section of the app was to provide additional value to the user
Over 65 simple tips 'micro-interventions', most based around mindfulness tips and activities were included and revealed in colourful fashion (See Blake 2014)

Icons

Simple icons on a white background
A text subtitle supports meaning
A circular form prompts interaction

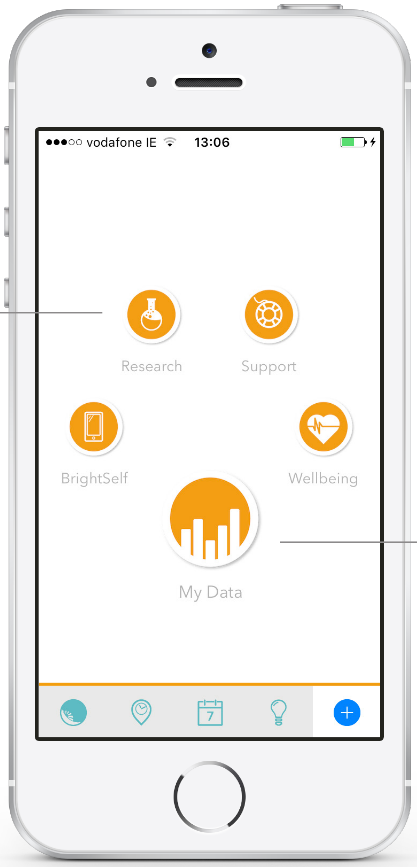


My Data

A larger icon indicates the relative value of this section
An orange colour scheme is used to denote all 'extras' components

EXTRAS SCREEN

Extras

Separate presentation of these components marks them as separate to the core content of the app

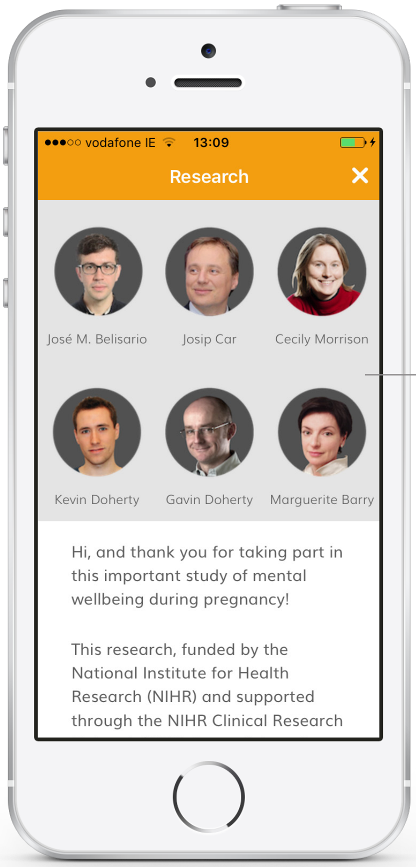


RESEARCH SCREEN

Researcher Photos

Images of the researchers were included in an attempt to establish trust

Research

The motivations behind this research, its funding sources and those involved Establishing users' trust, a working alliance, and emphasing users' contribution to science may support engagement

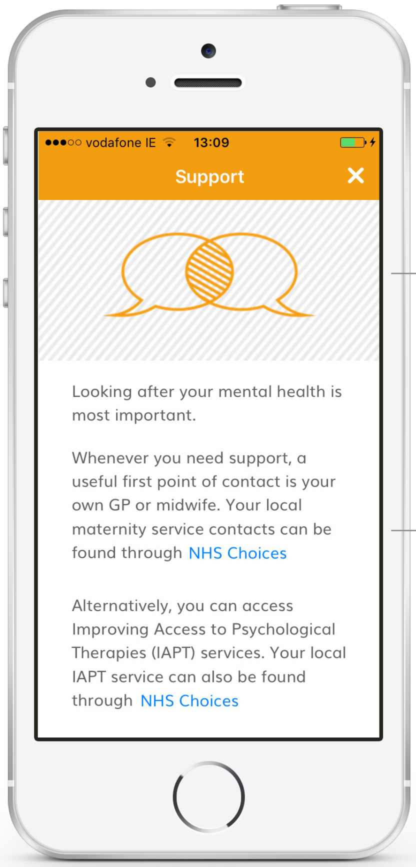


Opening image

Aesthetically Engaging

Support

Links to several NHS and Mental Health Charity Support options, each with images, web links and phone numbers

*``So even the links for the NHS can be just provided'' (Female Midwife)*

SUPPORT SCREEN

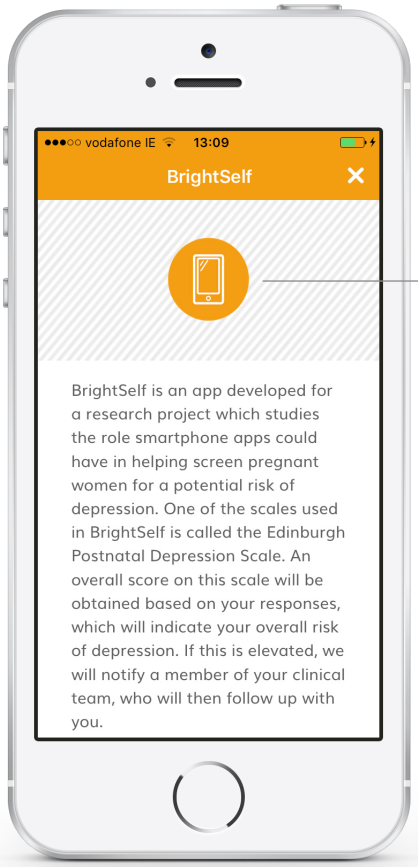


Opening image

Aesthetically Engaging

BRIGHTSELF SCREEN

BrightSelf

Information about the app, its content and features
Can be used to guide the user and also to introduce the app to others if needed

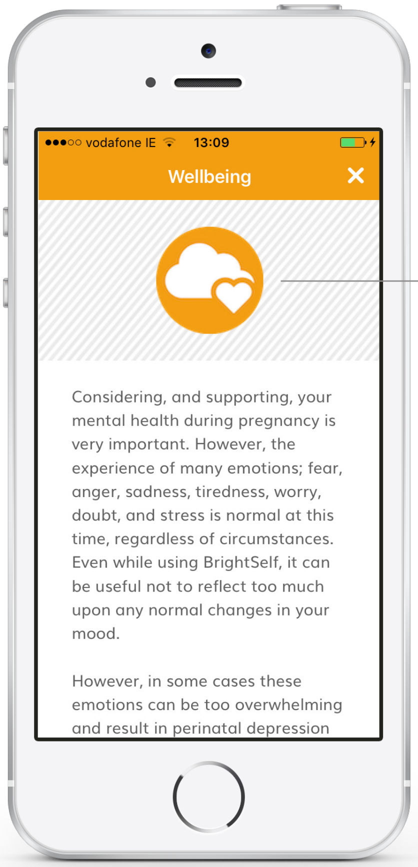


Opening image

Aesthetically Engaging

WELLBEING SCREEN

Wellbeing

Information about well-being during pregnancy, what is normal, and the importance of self-care

*``It's pretty rare that you get presented with the genuine full spectrum of things that you might be feeling, in a way which is kind of compelling'' (P6)*

## Day v Month

Data Display by Day or Month, and the ability to move between each time period

## CheckIn Display

The choice of CheckIn concept to display

## Graph

A minimal line graph showing individual reports and trends over time

## Extra Info

A popup provides an explanation of the chart and the data it draws on

## CheckBack Display

The option to display EPDS results
Displayed as a red bar for the 7-day period they cover
The CheckBack plot sits behind the CheckIn plot to facilitate comparison

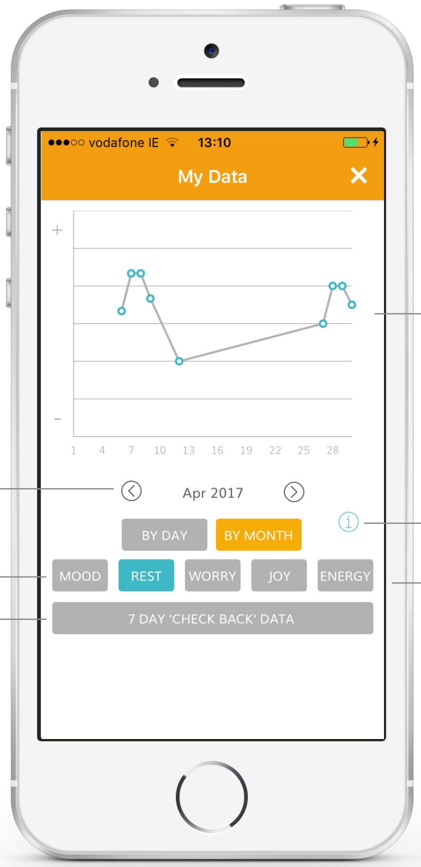


MY DATA SCREEN

## My Data

The user's results on all scales over time visualised

## Interaction

A simple symmetrical on/off interaction mechanism was chosen to facilitate the display and interpretation of this data
The colours of each button match the colour of the data displayed on the chart
